# Novel Ex Vivo Zymography Approach for Assessment of Protease Activity in Tissues with Activatable Antibodies

**DOI:** 10.3390/pharmaceutics13091390

**Published:** 2021-09-02

**Authors:** Bruce Howng, Michael B. Winter, Carol LePage, Irina Popova, Michael Krimm, Olga Vasiljeva

**Affiliations:** CytomX Therapeutics, Inc., San Francisco, CA 94080-1913, USA; bhowng@cytomx.com (B.H.); mwinter@cytomx.com (M.B.W.); clepage@cytomx.com (C.L.); irinakpopova@gmail.com (I.P.); mkrimm@cytomx.com (M.K.)

**Keywords:** protease activity, in situ zymography, Probody therapeutics, diagnostic, therapeutic, cancer

## Abstract

Proteases are involved in the control of numerous physiological processes, and their dysregulation has been identified in a wide range of pathologies, including cancer. Protease activity is normally tightly regulated post-translationally and therefore cannot be accurately estimated based on mRNA or protein expression alone. While several types of zymography approaches to estimate protease activity exist, there remains a need for a robust and reliable technique to measure protease activity in biological tissues. We present a novel quantitative ex vivo zymography (QZ) technology based on Probody^®^ therapeutics (Pb-Tx), a novel class of protease-activated cancer therapeutics that contain a substrate linker cleavable by tumor-associated proteases. This approach enables the measurement and comparison of protease activity in biological tissues via the detection of Pb-Tx activation. By exploiting substrate specificity and selectivity, cataloguing and differentiating protease activities is possible, with further refinement achieved using protease-specific inhibitors. Using the QZ assay and human tumor xenografts, patient tumor tissues, and patient plasma, we characterized protease activity in preclinical and clinical samples. The QZ assay offers the potential to increase our understanding of protease activity in tissues and inform diagnostic and therapeutic development for diseases, such as cancer, that are characterized by dysregulated proteolysis.

## 1. Introduction

Proteases, or proteolytic enzymes, catalyze the breakdown of proteins by hydrolysis of peptide bonds. More than 500 proteases (≈2% of the genome) have been identified using bioinformatic analysis of murine and human genomes [1,2] and can be categorized in five distinct classes based on their catalytic mechanisms: serine, cysteine, aspartic, metalloproteases, and threonine proteases [3]. Proteases are involved in the control of a multitude of key physiological processes, such as hemostasis [4], immunity [5], fertility [6], cell survival, proliferation and differentiation [7], and apoptosis [8].

Normally, protease activity is tightly regulated through multiple redundant mechanisms, including gene expression, zymogen activation, endogenous inhibitors, subcellular localization, and post-translational modifications [3]. Protease dysregulation has been identified in a wide range of pathologies, including cardiovascular, neurodegenerative and inflammatory diseases, infection, and cancer [2]. Notably, dysregulated proteolysis is central to carcinogenesis by playing key roles in tumor progression-associated processes, including growth, invasion, and metastasis [9,10,11]. Due to their involvement in multiple pathologies, proteases represent attractive biomarkers or drug targets in wide-ranging therapeutic areas, including cancer.

Protease expression levels can be measured using mRNA quantification, proteomics, or by immunoassays, such as immunohistochemistry (IHC) or enzyme-linked immunosorbent assays (ELISAs). However, because proteases are regulated by multiple post-translational mechanisms, mRNA expression and immunoassays are not necessarily predictive of protease activity levels. For example, mRNA expression and immunoassays are unable to distinguish between active and inactive zymogen forms of proteases or those complexed with endogenous protease inhibitors [12].

In contrast, zymography, which relies upon the visualization of enzymatic substrate conversion, enables the direct measurement of protease activity through the detection of cleavage product formation, or alternatively, substrate depletion [12]. The combined use of molecular weight separation and zymography in the in-gel zymography approach provides qualitative, as well as quantitative, information and allows for the differentiation of intact, activated, and complexed proteases. However, the tissue homogenization process often utilized for in-gel zymography may allow aberrant ex vivo proteolysis of substrates, which would impact the assay outcome [13]. In addition, proteases can be denatured during the electrophoresis process [14]. While in situ zymography of tissue sections obviates these risks, these approaches generally rely on broad-spectrum, dye-quenched protein substrates such as DQ-gelatin, DQ-collagen I, and DQ-collagen IV [15,16]. Recently, in situ zymography approaches utilizing more selective protease substrates have been reported based on targeted, internally quenched nanosensors or complexed poly-arginine peptides [17,18]. Additionally, an important advancement in this field was recently achieved by Poreba et al. [19] through the development of a novel approach based on active site probes and CyTOF methodology, which enabled the profiling of a subset of intracellular proteases in cancer cell lines and PBMC cell populations [19]. There is also a potential for synchronous evaluation of multiple proteases using active site-specific, anti-protease antibodies by IHC methods [20,21,22,23]. Therefore, several types of zymography approaches exist, but there remains an outstanding need for a robust, reliable, and widely applicable technique to estimate physiological and pathological protease activity, especially in tissue samples.

Several protease activity-based diagnostic probes [17,18,24,25,26,27,28,29,30] and therapeutics [31,32,33,34] have been recently developed by exploiting the characteristically dysregulated protease activity seen in cancer [11,35]. For example, Probody^®^ therapeutic(s) (Pb-Tx) are a novel class of conditionally activated therapeutics consisting of a tumor-targeting antibody (or antibody fragment) that is masked by a peptide blocking the antigen-binding domain that is tethered via a protease-cleavable linker [31,36,37,38,39]. Therefore, the conditional activation of a Pb-Tx by proteases at the tumor site can improve tumor specificity and reduce off-tumor/on-target toxicity [36,40,41,42]. We previously reported an IHZ technology based on the unique features of a Pb-Tx and IHC, which can be applied to profile and monitor protease activity in any biological tissue [43]. This assay is based on Pb-Tx binding to the target upon activation by tumor-associated proteases and thus requires target expression for the visualization of protease activity. Here, we present an improved version of the assay, which is based on Pb-Tx technology and capillary electrophoresis and does not require target binding for protease activity assessment.

## 2. Materials and Methods

### 2.1. Biological Materials

Recombinant human membrane-type serine protease 1 (MT-SP1) (3946-SEB), urokinase-type plasminogen activator (uPA) (1310-SE), and matrix metalloproteinase-2 (MMP-2) (902-MP) were from R&D Systems (Minneapolis, MN, USA). Human tumor samples were provided by the NCI Cooperative Human Tissue Network; other investigators may have received samples from the same tissue specimens. The H292 cell line (CRL-1848) for xenograft studies was acquired from the American Type Culture Collection. Plasma samples were obtained from ProteoGenex (Inglewood, CA, USA).

### 2.2. Pb-Tx Labeling

The methods for the production of Pb-Tx have been described previously [43]. Pb-Tx for assessment of tumor sections were conjugated with the far-red fluorescent Alexa Fluor 647 dye (A20006, ThermoFisher Scientific, Waltham, MA, USA) using *N*-hydroysuccinimide ester reaction. Pb-Tx were incubated with amine-reactive fluorescent dyes for 1 h at room temperature, and the reaction was stopped with 1 M tris(hydroxymethyl)aminomethane hydrochloride (Tris-HCl) buffer, pH 8.5. After conjugation, free dye was removed using Zeba desalting columns (87,768, ThermoFisher Scientific), according to manufacturer protocols. Pb-Tx with a degree of labeling of 2 to 3, as determined by a NanoDrop spectrophotometer (ThermoFisher Scientific), were used. Pb-Tx for assessment of plasma samples were conjugated with Oregon Green dye (O6149, ThermoFisher Scientific).

### 2.3. QZ Assay Protocol

Assessment of activity in tumor sections using the QZ assay was performed on frozen tissue sections that had been stored at −80 °C for long-term storage and at −20 °C shortly before use. Slides were brought to room temperature and allowed to dry for 30 min before the assay. A hydrophobic barrier was drawn around the tissue sample to maintain liquid on the tissue using an ImmEdge Hydrophobic Barrier Pen (Vector Laboratories, Burlingame, CA, USA), and then, the slides were incubated with buffer consisting of 150 mM Tris HCl pH 7.4, 5 mM CaCl_2_ 100 µM ZnCl_2_, and 0.005% Tween-20 (QZ assay buffer) for 30 min at room temperature. Then, labeled Pb-Tx prepared in QZ buffer were added directly onto the tissue containing the buffer and incubated at a concentration of 20 µg/mL in a humidified chamber at 37 °C for 48 h. For inhibitor treatment assays utilizing binding-competent C225 Pb-Tx, tissue sections were blocked for 30 min with 3X unlabeled C225 antibody (60 µg/mL) and then preincubated for at least 30 min with 3X protease inhibitors before the addition of 3X labeled C225 Pb-Tx (60 µg/mL) in QZ buffer. Protease inhibitors and their final (1X) assay concentrations were as follows: 200 µg/mL aprotinin (78432, ThermoFisher Scientific), 10 µM Galardin or GM 6001 (364,206, Calbiochem, Burlington, MA, USA), and 1X Halt Protease Inhibitor Cocktail (78,438, ThermoFisher Scientific).

Following 48 h of incubation, conditioned media from samples were collected into 96-well PCR plates. Samples were mixed with Pico Sample Buffer (Perkin Elmer, Waltham, MA, USA) containing 2-beta-mercaptoethanol at four parts sample and one part of Pico Sample Buffer and then heated at 95 °C for 10 min. Then, Pb-Tx activation was assessed using the Caliper LabChip GXII or LabChip GXII Touch (Caliper Life Sciences, Waltham, MA, USA) with the HT Pico Protein Express 100 protocol (Perkin Elmer). Protein Express Assay LabChips (Perkin Elmer #760,499) were set up using the protocol of the Protein Pico Assay Reagent Kit (Perkin Elmer #760,498). Activation of the Pb-Tx was assessed on LabChip GX Reviewer software (Perkin Elmer), with relative activation determined by dividing the activated light chain peak area by the total activated and intact light chain peak area.

### 2.4. In Vivo Efficacy Study

Eight-week-old female Fox Chase severe combined immunodeficiency mice (Charles River Laboratories, Wilmington, MA, USA) were implanted subcutaneously in the right hind flank with 5 × 10^6^ H292 cells. The implant medium contained a 1:1 mixture of serum-free RPMI media and Matrigel (Corning, Corning, NY, USA). After tumors were palpable, body weights and tumor volumes were collected twice weekly. Tumors were measured using digital calipers and tumor volumes were calculated using the formula: tumor length × (tumor width)^2^ × 0.52 = tumor volume. Eleven days after tumor cell implantation, mice were assigned to treatment groups of eight mice each. Each treatment group had an approximately equal mean tumor volume of 140 mm^3^. After assignment, mice were treated with a single 10 mL/kg intraperitoneal dose of the test article.

### 2.5. QZ Assay of Human Plasma Samples to Assess the Stability of CX-188 in Plasma In Vitro

Oregon Green-labeled Pb-Tx in phosphate-buffered saline (PBS), pH 7.2, were added to an equal volume of plasma from healthy donors (*n* = 5) or patients with lung cancer (*n* = 5) or gastric cancer (*n* = 5) (ProteoGenex, Inglewood, CA, USA) to give a final concentration of 714 µg/mL in a total volume of 10 µL. The Pb-Tx and plasma mixtures were incubated for 48 h at 37 °C in a humidified chamber; then, they were diluted 1:50 in PBS and analyzed with Western capillary electrophoresis (Wes). Samples were separated for 33 min to resolve activated and intact Pb-Tx and then incubated for 1 h with goat polyclonal antibodies against Oregon Green Dye (1/40; A11095 ThermoFisher). Target proteins were immunoprobed using a 30 min incubation with a horseradish peroxidase-conjugated secondary antibody (1/40; 705-035-147, Jackson ImmunoResearch, West Grove, PA, USA) and chemiluminescent substrate (PS-CS0; ProteinSimple Cat, San Jose, CA, USA). The activation of the labeled Pb-Tx was calculated from the chemiluminescent signals of the activated and intact light chain, as measured using the integrated Simple Western Compass software (ProteinSimple). Only peaks with a signal-to-noise ratio of ≥5 were considered positive. This signal-to-noise ratio was calculated by the Simple Western Compass software (ProteinSimple).

### 2.6. Statistical Analysis

Data are presented as mean ± standard error. Statistical significance was analyzed using the two-sided Student’s *t*-test (*p* ≤ 0.05 was considered statistically significant).

## 3. Results

### 3.1. Quantitative Zymography (QZ) Technology for Assessment of Protease Activity Ex Vivo

Pb-Tx consist of an active antibody (or antibody fragment), a prodomain containing a unique masking peptide, and a protease-cleavable substrate linker. The mask, identified by screening a peptide library [44], is fused to the amino terminus of the antibody light chain and blocks antibody binding. The linker between the mask and antibody contains a substrate that is cleaved by selected protease classes. Protease-specific substrates are identified by screening a peptide library using a tagged bacterial display system that enables positive and negative selection by fluorescence-activated cell sorting of peptides cleaved by recombinant proteases of interest [45].

**Figure 1:**
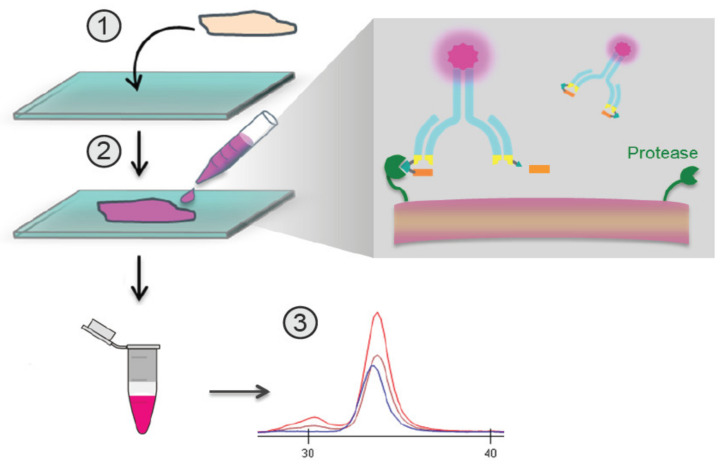
Overview of the in situ quantitative zymography (QZ) assay. Schematic demonstrating QZ methodology for the assessment of in situ Probody therapeutic(s) (Pb-Tx) activation. Labeled Pb-Tx are incubated with a tissue section, and the extent of Pb-Tx activation is measured in conditioned medium using capillary electrophoresis (CE).

**Figure 2:**
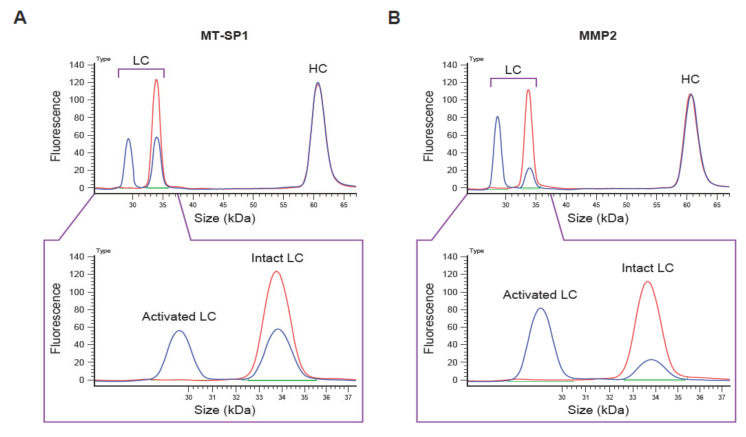
Example of capillary electrophoresis (CE)-based Pb-Tx cleavage assessment. Electropherograms of CE-based cleavage assessment of the C225-Sub1 Pb-Tx incubated with (**A**) recombinant human membrane-type serine protease 1 (MT-SP1) and (**B**) matrix metalloproteinase-2 (MMP-2) for 4 h. LC, light chain; HC, heavy chain; Pb-Tx, Probody therapeutic.

The QZ technique described here involves the incubation of labeled Pb-Tx with a tissue sample (Figure 1). If the sample includes proteases that activate the Pb-Tx, the substrate will be cleaved, leading to dissociation of the prodomain, which can be measured in conditioned medium by capillary electrophoresis (Figure 2). By generating Pb-Tx with differing protease specificities, the profile of specific protease activities can be characterized in a semi-quantitative manner.

To avoid potential binding of the activated Pb-Tx to the antigen, a non-binding antibody was designed by several mutations of the C225 anti-epidermal growth factor receptor (EGFR) antibody heavy chain (HC) complementarity-determining regions (Figure S1). The resulting mutant antibody (MC225) was not capable of binding to the EGFR antigen and thus serves as a universal reagent for the QZ assay (Figure S1).

### 3.2. Optimization of QZ Assay Conditions

**Figure 3:**
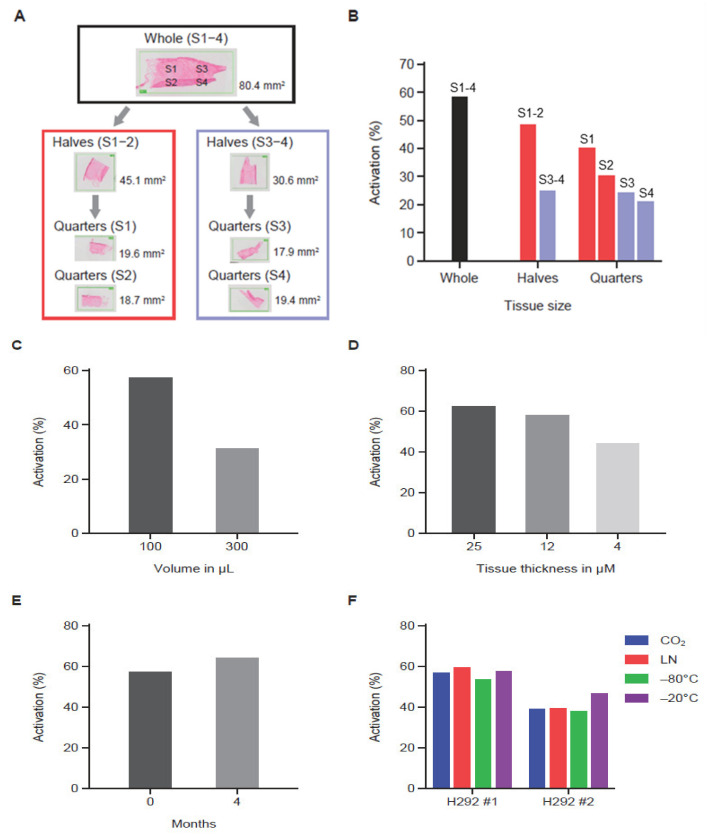
The effect of experimental variables on the quantitative zymography (QZ) assay. (**A**) Diagram showing tissue partitioning into halves and quarters prior to QZ activity assessment. (**B**) QZ assay of a tissue sample assessed at different section sizes using a constant assay volume (100 µL). As the tissue was halved and quartered, decreased activity was observed. (**C**) QZ assay of a tissue sample assessed in different assay volumes (100 µL and 300 µL) showing correlation of assay volume with activity. (**D**) QZ assay of tissue sections of different thickness in a fixed volume showing correlation of tissue thickness with activity. (**E**) QZ assay before and after tissue storage at −80 °C for 4 months showing preservation of activity after storage. (**F**) QZ assay of tissue samples subjected to different freezing techniques and storage temperatures. Samples frozen using dry ice (CO_2_), liquid nitrogen (LN), or −80 °C temperature were stored at −80 °C. Samples frozen using −20 °C temperature were stored at −20 °C. Activity was similar regardless of freezing condition and storage temperature. For data shown in panels (**B**–**E**), the Sub1 Probody therapeutic (Pb-Tx) was used, while for panel (**F**), the Sub2 Pb-Tx was used. The incubation time for data shown in panels (**B**–**D**) was 24 h. The incubation time was 48 h for data shown in panels (**E**,**F**).

MC225 Pb-Tx containing protease substrates Sub1 or Sub2 were used to assess the effect of experimental variables on QZ activity. Here, archival bladder cancer tissue from patients or H292 human non-small cell lung cancer xenograft tumor sections were utilized. Three human tumor serial sections were analyzed by leaving one whole and dividing the other two into halves or quarters for tumor tissue correlation studies (Figure 3A). Using the MC225 Sub1 Pb-Tx, the level of protease-activated Pb-Tx was found to be proportional to tissue content in the tissue halves; however, the quarters showed some variability (Figure 3B), indicating some degree of protease activity heterogeneity across the tumor sample. Next, QZ activity assessment of tumor sections of the same size but with different assay buffer volumes supported the correlation of activated Pb-Tx levels with the relative concentration of protease activity (Figure 3C). Furthermore, the effect of tumor section thickness was also explored, demonstrating that the Pb-Tx activation signal decreases with reduced section thickness (Figure 3D). Protease zymography assays are performed on fresh or frozen tissues that maintain endogenous protease activity; therefore, conditions of tissue cryopreservation and storage were extensively evaluated. Specifically, assessment of protease activity in fresh compared to frozen and stored tumor tissue samples showed that Pb-Tx activation did not decrease with frozen storage at −80 °C for 4 months (Figure 3E). Similarly, Pb-Tx activation was unaffected by freezing technique (liquid nitrogen or carbon dioxide) or temperature (−80 °C or −20 °C; Figure 3F). The technical reproducibility of the assay was confirmed in H292 xenograft tumor samples and patient tissues (Figure S2).

### 3.3. Assessment of Protease Activity of Different Specificities

**Figure 4:**
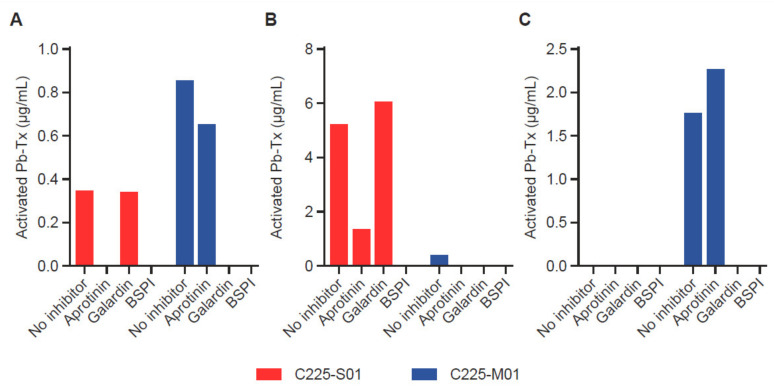
Validation of the quantitative zymography (QZ) assay for different protease specificities in human tumor tissue. Serine protease and matrix metalloproteinase (MMP) activity were measured with 20 µg/mL Probody therapeutics (Pb-Tx) C225-S01 and C225-M01, respectively, after incubation for 48 h at 37 °C with tissue sections. Protease activity was characterized in cryopreserved sections of (**A**) head and neck squamous cell carcinoma, (**B**) pancreatic cancer, and (**C**) prostate cancer in the presence of the following protease inhibitors: serine protease inhibitor aprotinin, MMP protease inhibitor Galardin, and a broad-spectrum protease inhibitor cocktail (BSPI).

To demonstrate the ability of the QZ assay to assess the activity of proteases with different specificities, we utilized Pb-Tx containing a serine protease substrate (LSGRSDNH, C225-S01) cleavable by at least two serine proteases, MT-SP1 and urokinase-type plasminogen activator (uPA), and a broad-spectrum matrix metalloproteinase (MMP)-specific substrate (PLGL, C225-M01) (Figure S3). To evaluate the heterogeneity of protease activity in tumor tissue, we measured the activation of C225-S01 and C225-M01 Pb-Tx in tumor samples from patients with different types of cancer: head and neck squamous cell carcinoma (HNSCC), pancreatic cancer, and prostate cancer (Figure 4A–C). Protease inhibitors were used to confirm the specificity of the protease activity measured by both Pb-Tx. Notably, the HNSCC tumor sample revealed the presence of both serine and MMP protease activity. As expected, the activation signal of both C225-S01 and C225-M01 Pb-Tx was inhibited by pre-treatment of the tissues with a broad-spectrum protease inhibitor cocktail. However, whereas C225-S01 activation was abolished by the serine protease-specific inhibitor aprotinin and not the MMP-specific inhibitor Galardin, a reverse inhibition pattern was detected for C225-M01. Thus, these data demonstrate that the QZ assay can differentiate between different protease specificities by exploiting substrate specificity and selectivity and that further refinement can be achieved using protease-specific inhibitors. Additional tissue samples of human pancreatic and prostate tumors were identified as examples of tissues with predominant serine or MMP protease activity, respectively, using the Pb-Tx and protease class-specific inhibitors (Figure 3C and Figure 4B). Furthermore, to compare the QZ assay to a traditional fluorescent probe approach, we have determined that the *k*_cat_/*K*_m_ values of substrate S01 in the context of an internally quenched (IQ) probe versus a Pb-Tx for uPA and MT-SP1 were 4.6 and 3.5 times higher, respectively (Figure S4), thus additionally supporting the translational value of the QZ assay for the development of conditionally activated therapeutics.

### 3.4. QZ Technology for Protease Characterization in H292 Xenograft Tumor Sections

**Figure 5:**
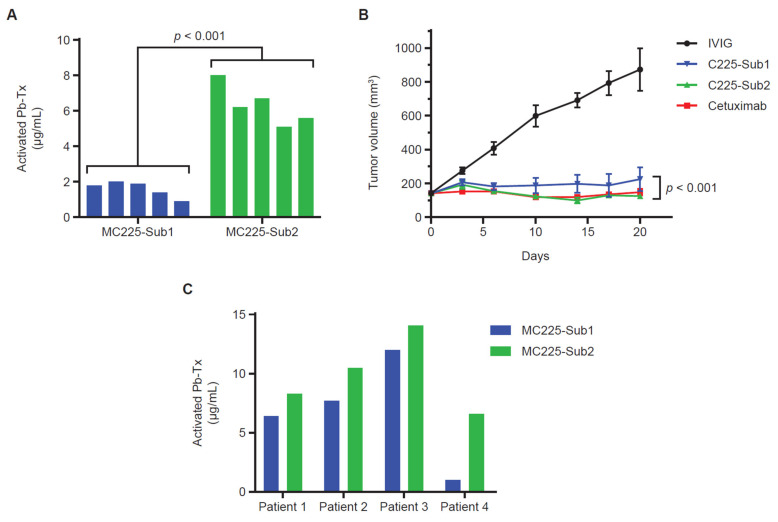
Sub1 vs. Sub2 activation in xenograft models and human tumor tissue. (**A**) Quantitative zymography (QZ) assessment of C225 Probody therapeutics (Pb-Tx) activation with H292 cell line-derived xenograft (CDX) tissue. A total of 20 µg/mL of MC225-Sub1 or MC225-Sub2 was incubated for 48 h at 37 °C with tissue sections from H292 CDX tumors (*n* = 5). (**B**) In vivo efficacy at 10 mg/kg (mpk) of the C225 Pb-Tx with Sub1 or Sub2 substrates compared to cetuximab and intravenous immunoglobulin therapy (IVIG) in the H292 xenograft model (*n* = 8 mice per group). (**C**) QZ assessment of MC225-Sub1 and MC225-Sub2 Pb-Tx activation with cholangiocarcinoma patient tissues (*n* = 4). Statistical significance was calculated by Student’s *t*-test.

To further investigate the correlation of protease activity measured in situ with the QZ assay and in vivo Pb-Tx activity, we utilized the EGFR-responsive H292 xenograft model. For these experiments, Pb-Tx containing the multi-specific protease substrates Sub1 and Sub2 were utilized. Based on the substrate designs, MC225 Sub1 is less cleavable by selected MMP proteases than MC225 Sub2 and therefore demonstrated a lower activation rate in situ (Figure 5A). This finding was consistent across several H292 xenograft tumors from different mice (*n* = 5). Notably, this in situ activation profile was corroborated by Pb-Tx in vivo efficacy in the H292 xenograft tumor model (Figure 5B). While both the C225 Sub1 and C225 Sub2 Pb-Tx demonstrated significant efficacy at the 10-mg/kg dose, efficacy was enhanced with C225 Sub2 compared with C225 Sub1. These data demonstrate translation of the in situ QZ activation findings to the biological activity of Pb-Tx in in vivo models and highlight a potential role for the QZ assay in the prediction of protease substrate activation in biological systems. We further assessed the activation of these Pb-Tx in tissue samples from four patients with cholangiocarcinoma (Figure 5C). Consistent with the xenograft tumor results, a slightly higher activation rate was observed with MC225 Sub2 compared with MC255 Sub1. These results further demonstrate that the QZ technology enables the assessment of protease activity in patient tissues and supports the design of specific protease substrates that leverage the activities of tumor-associated proteases for therapeutic activation.

### 3.5. QZ Technology for Assessment of Protease Activity in Patient-Derived Plasma Samples

The QZ assay can be used for the detection of protease activity in liquid as well as in solid biological samples. Here, we present an example of the assessment of a programmed cell death 1 (PD-1) Pb-Tx (CX-188) in the plasma of heathy donors and patients with cancer. We first established that a capillary electrophoresis immunoassay-based method effectively differentiated the activated and intact CX-188 Pb-Tx. The lower limit of detection of activated CX-188 was determined to be ≥1 µg/mL. After 48 h of incubation of CX-188 in plasma samples from normal healthy volunteers (n = 5) and patients with lung (*n* = 5) or gastric cancer (*n* = 5), there was no evidence of CX-188 cleavage (Tables S1–S3, Figure S5), demonstrating that the Pb-Tx are stable in patients’ plasma as designed.

## 4. Discussion

Proteases are increasingly recognized as integral regulatory components of numerous biological mechanisms. Therefore, there is increasing interest in the potential utility of proteases as biomarkers for diagnostics and targets for therapeutics in a wide range of pathological conditions [2]. Consequently, reliable and robust methods are required to better understand the physiological and pathological relevance of proteases and their potential roles in the management of disease. As mRNA and protein expression assays are generally unable to distinguish zymogens and active and degraded enzymes, these assays are inappropriate for accurate estimation of protease activity. Some novel zymography assays allow the evaluation of physiological and pathological protease activity, but zymography remains a relatively immature field compared with protein and gene expression [12]. Consequently, there remains an outstanding need for effective, reliable, and reproducible methods for the estimation of functional protease activity in biological samples.

Representing a novel in situ zymography approach, the results reported here demonstrate that the QZ assay enables the detection of specific protease activity in preclinical and clinical samples. After exposure of a labeled, protease-activatable Pb-Tx to the sample, endogenous protease activity can be assessed by capillary electrophoresis to identify intact and cleaved Pb-Tx levels. Earlier in situ zymography techniques, which relied on the direct visualization of fluorescein-tagged substrates, commonly used non-specific substrates, such as DQ collagen [15]. Furthermore, while active site-specific anti-protease antibodies have been investigated, they are limited to a few antibodies for a small subset of proteases [20,21,22,23]. In contrast, the QZ assay can be adapted to virtually all proteases, provided that selective substrates and the optimal conditions for individual proteases are available and applied. In cases in which substrates are not sufficiently selective to distinguish between individual proteases due to overlapping protease specificities, the QZ assay can be complemented with the use of inhibitors, individually or in combination, to characterize the different protease activity levels.

In contrast to an earlier Pb-Tx-based in situ zymography technique we reported, called IHZ, which relies on the detection of activated Pb-Tx binding to the target [43], the QZ assay leverages capillary electrophoresis for the detection of activated Pb-Tx in a target-agnostic manner. Therefore, the QZ assay more specifically measures protease activity compared with the IHZ assay, which addresses therapeutic activation and target binding. Furthermore, IHC-based techniques have long been associated with multiple challenges in staining and scoring, although these have been largely addressed by digital IHC analysis, which was introduced to overcome these limitations and allow high-resolution, high-contrast, whole-slide imaging [46]. Conversely, capillary electrophoresis gained considerable interest in the field of study of monoclonal antibodies due to its high resolution and efficiency of separation of monoclonal antibodies and their derivatives [47,48], and it offers rapid, reproducible, and objective assessment that does not require antigen binding for signal detection. Other in situ zymography approaches have been introduced utilizing a quenched protease substrate conjugated to an integrin-targeted nanoparticle [17] or contained within a complexed poly-arginine probe for cell surface staining upon activation [49]. In contrast to QZ, these approaches are also limited by the requirement for target engagement or cell surface labeling for fluorescence-based staining detection of protease activity.

To interrogate the effects of specific assay variables on QZ activation, an extensive evaluation of assay conditions was performed. These data indicate that variables, such as tissue size and assay volume, can affect the activation readout and that controlling for these factors enables more direct comparison between samples. Of note, protease activity was not affected by prolonged sample storage at −80 °C (Figure 3E) and did not vary substantially when different freezing techniques and temperatures were utilized (Figure 3F), suggesting that the assay can be widely used across different laboratories and is applicable for the evaluation of archival as well as fresh tumor tissue. In addition, the QZ assay was shown to be applicable to the interrogation of protease activity in liquid biospecimens. As such, we have applied the QZ assay principle to evaluate protease activity in plasma of healthy donors and cancer patients. No activation of Pb-Tx was detected in patient plasma samples, which is consistent with the specificity of Pb-Tx for tumor-associated proteases and clinical data, demonstrating that Pb-Tx are activated in tumor sites but remain predominantly intact in plasma in vivo [50]. Notably, protease activity is expected to be low in plasma in vitro due to the abundance of endogenous protease inhibitors that represent nearly 10% of the total protein in plasma and by weight, constitute the third largest group of functional proteins after albumin and the immunoglobulins [51,52]. Together, these findings suggest that the QZ assay represents a robust protease activity measuring technology that may have expanded utility compared with IHC-based methods.

Assessment of protease activity in H292 tumor xenograft tissue demonstrated that Pb-Tx molecules differing only in protease substrate were differentially activated by xenograft tumors derived from a single tumor cell line (Figure 5A). QZ-assessed protease activity was increased with the Sub2 substrate compared with the Sub1 substrate due to the specific design elements of the former substrate associated with increased cleavability by MMP proteases. Furthermore, an in vivo study assessing Pb-Tx with Sub1 and Sub2 showed increased efficacy for C225 Sub2 compared to C225 Sub1 (Figure 5B), thus agreeing with the in situ QZ assessment of these two constructs. These findings demonstrate that the QZ assay can be predictive of in vivo activity and can differentiate between substrates with different protease cleavability profiles.

The application of the QZ technique to the evaluation of clinical tissue specimens was also assessed and showed that the assay was suitable for the measurement of protease activity in patient tumor samples (Figure 4 and Figure 5C). Evaluation of human tumor tissue sections demonstrated the presence of detectable protease activity in most tumors across multiple indications. Moreover, the QZ assay can also be used for cataloging and differentiating protease specificity in human tissue with the use of more selective substrates and/or specific protease inhibitors.

The current Pb-Tx technology has been used to develop a range of conditionally activated therapeutics with a diverse profile of target antigens and demonstrated clinical benefit [36,41,42]. Pb-Tx-based assays have been used to inform therapeutic development and further validate the Pb-Tx platform [39,50]. Notably, the cleavability of a given protease substrate can be affected by the scaffold and format of the probe. For example, the increased cleavability of a conventional small peptide probe was observed compared to a Pb-Tx containing the same substrate, which is likely due to differences in protease accessibility (Figure S4). Therefore, the assessment of substrate activation in biological tissues using surrogate small molecules or fluorescent probes can potentially overestimate the degree of substrate cleavage and might have limited predictive value for the development of conditionally activated therapeutics.

Taken together, the current study shows that the QZ assay is a robust and reliable method for in situ assessment of protease activity in clinical, as well as in preclinical, samples and has the potential to be applied to a wide range of clinical sample types, including plasma, cerebrospinal fluid, bone marrow, synovial fluid, and tissue samples. With the ability to tailor Pb-Tx substrate specificity for diverse proteases, the QZ assay offers the potential to enhance our current understanding of the role of proteases in numerous biological processes. The assay is also well positioned to inform the development of therapeutics for the wide range of diseases in which dysregulated protease activity has been implicated.

## Data Availability

All data relevant to the study are included in the article or uploaded as online supplemental information. The datasets used and/or analyzed during the current study are available from the corresponding author on reasonable request.

## Supplementary Materials

The following are available online at https://www.mdpi.com/article/10.3390/pharmaceutics13091390/s1, Table S1: In vitro stability of CX-188 in plasma from healthy human donors, Table S2: In vitro stability of CX-188 in plasma from lung cancer patients, Table S3: In vitro stability of CX-188 in plasma from gastric cancer patients, Figure S1: Design of non-binding antibody, Figure S2: Reproducibility of the assay, Figure S3: C225-M01 and C225-S01 Pb-Tx protease selectivity, Figure S4: Substrate protease cleavability in an internally quenched (IQ) probe vs. Pb-Tx format, Figure S5: Electropherogram images of CX-188 incubated in plasma.
