# Supplementary material for: Novel Ex Vivo Zymography Approach for Assessment of Protease Activity in Tissues with Activatable Antibodies
Source: Pharmaceutics. 2021 Sep 2;13(9):1390. doi: 10.3390/pharmaceutics13091390 (PMC8471274; doi:10.3390/pharmaceutics13091390)
Supplement: Supplementary file 1 [file pharmaceutics-13-01390-s001.zip › pharmaceutics-1273372 - supplementary material.pdf]

pharmaceutics

MDPI

Supplementary Material: Novel Ex Vivo Zymography Approach for Assessment of Protease Activity in Tissues with Activatable Antibodies

Bruce Howng, Michael B. Winter, Carol LePage, Irina Popova, Michael Krimm and Olga Vasiljeva

Table S1. In Vitro Stability of CX-188 in Plasma from Healthy Human Donors.

Sample No.	Clinical Diagnosis	Location	Stage	Probody Activation
01	Normal	N/A	N/A	No activation detected
02	Normal	N/A	N/A	No activation detected
03	Normal	N/A	N/A	No activation detected
04	Normal	N/A	N/A	No activation detected
05	Normal	N/A	N/A	No activation detected

N/A = not applicable.

Table S2. In Vitro Stability of CX-188 in Plasma from Lung Cancer Patients.

Sample No.	Clinical Diagnosis	Location	Stage	Probody Activation
06	Lung cancer	Upper lobe of left lung	IIB	No activation detected
07	Lung cancer	Upper lobe of right lung	IB	No activation detected
08	Lung cancer	Lower lobe of left lung	IB	No activation detected
09	Lung cancer	Upper lobe of right lung	IIB	No activation detected
10	Lung cancer	Lower lobe of right lung	IB	No activation detected

Table S3. In Vitro Stability of CX-188 in Plasma from Gastric Cancer Patients.

Sample No.	Clinical Diagnosis	Histological Diagnosis	Grade	Stage	Probody Activation
11	Stomach cancer	Adenocarcinoma	G3	IIA	No activation detected
12	Stomach cancer	Adenocarcinoma	G2	IB	No activation detected
13	Stomach cancer	Adenocarcinoma and signet ring cell carcinoma	G2-3	IIIB	No activation detected
14	Stomach cancer	Adenocarcinoma	G2	IIA	No activation detected
15	Stomach cancer	Adenocarcinoma	G2-3	IIA	No activation detected

A

```
>Cetuximab_HC (hIgG1)
QVQLKQSGPGLVQPSQSLSITCTVSGFSLTNYGVHWVRQSPGKGLEWLGVIWSGGNTDYN
TPFTSRLSINKDNSKSQVFFKMNSLQSNDTAIYYCARALTYYDYEFAYWGQGTLVTVSAA
STKGPSVFPLAPSSKSTSGGTAALGCLVKDYFPEPVTVSWNSGALTSGVHTFPAVLQSSG
LYSLSSVVTVPSSSLGTQTYICNVNHKPSNTKVDKRVEPKSCDKTHTCPPCPAPELL
GGPSVFLFPPKPKDTLMISRTPEVTCVVVDVSHEDPEVKFNWYVDGVEVHNAKTKPREEQ
YNSTYRVVSVLTVLHQDWLNGKEYKCKVSNKALPAPIEKTISKAKGQPREPQVYTLPPSR
DELTKNQVSLTCLVKGFYPSDIAVEWESNGQPENNYKTTPPVLDSDGSFFLYSKLTVDKS
RWQQGNVFSCSVMHEALHNHYTQKSLSLSPGK

>Cetuximab_HC_mutant (hIgG1)
QVQLKQSGPGLVQPSQSLSITCTVSGFSLTSYGVHWVRQSPGKGLEWLGVIWSKGSTAYN
TPFTSRLSINKDNSKSQVFFKMNSLQSNDTAIYYCARALTSGKAEFAYWGQGTLVTVSAA
STKGPSVFPLAPSSKSTSGGTAALGCLVKDYFPEPVTVSWNSGALTSGVHTFPAVLQSSG
LYSLSSVVTVPSSSLGTQTYICNVNHKPSNTKVDKRVEPKSCDKTHTCPPCPAPELL
GGPSVFLFPPKPKDTLMISRTPEVTCVVVDVSHEDPEVKFNWYVDGVEVHNAKTKPREEQ
YNSTYRVVSVLTVLHQDWLNGKEYKCKVSNKALPAPIEKTISKAKGQPREPQVYTLPPSR
DELTKNQVSLTCLVKGFYPSDIAVEWESNGQPENNYKTTPPVLDSDGSFFLYSKLTVDKS
RWQQGNVFSCSVMHEALHNHYTQKSLSLSPGK
```

B

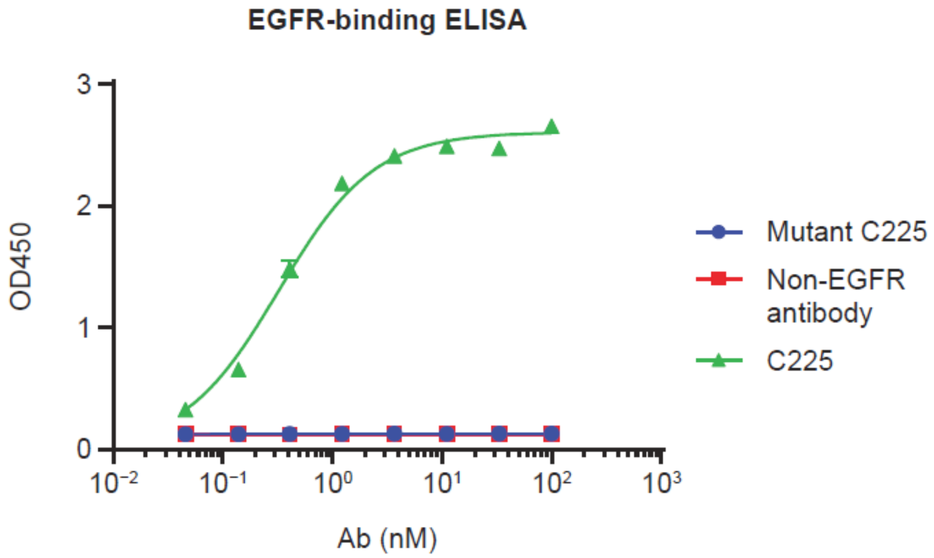

Figure S1. Design of non-binding antibody. (A) A non-binding antibody was designed by several mutations of the C225 anti-epidermal growth factor receptor (EGFR) antibody heavy chain (HC) complementarity-determining region. (B) The resulting mutant antibody (MC225) was not capable of binding to the EGFR antigen, as assessed by enzyme-linked immunosorbent assay. hIgG, human immunoglobulin G.

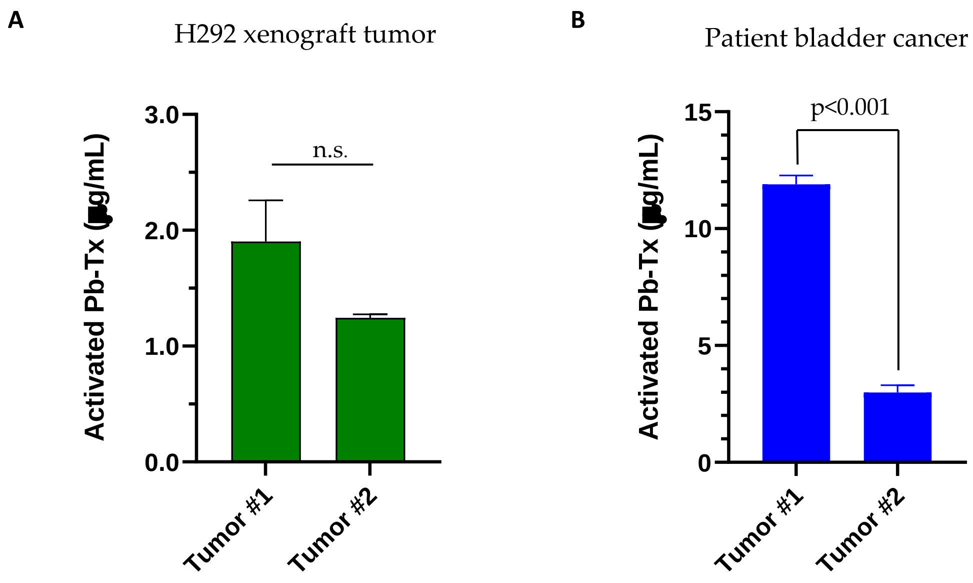

Figure S2. Reproducibility of the assay. Quantitative zymography (QZ) assessment of C225-Sub1 Pb-Tx activation in serial sections of H292 cell-line-derived xenograft (n = 2) and patient bladder cancer (n = 4) tumor samples. Data are presented as the mean and standard error. P values determined by the two-sided Student's t-test are shown. These data support reproducibility of the assay between different tissue sections of the same tumor sample and independent rounds of the assay. As expected, more variability is detected between different patients' tumors; however, the data from two tumors of the same mouse xenograft model demonstrate similar protease activity.

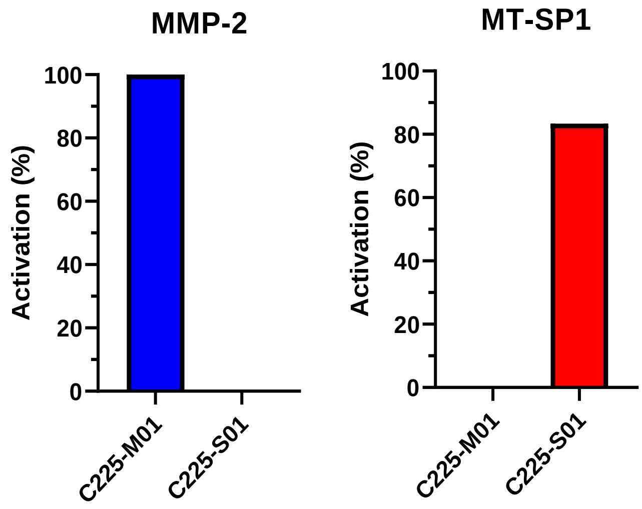

Figure S3. C225-M01 and C225-S01 Pb-Tx protease selectivity. Pb-Tx cleavage assessment demonstrating the matrix metalloproteinase (MMP) or serine protease class selectivity of C225-M01 and C225-S01, respectively. Cleavability was tested using recombinant human matrix metalloproteinase-2 (MMP-2) (4h) and membrane-type serine protease 1 (MT-SP1) (24h), and percent activation was measured by capillary electrophoresis (CE). .

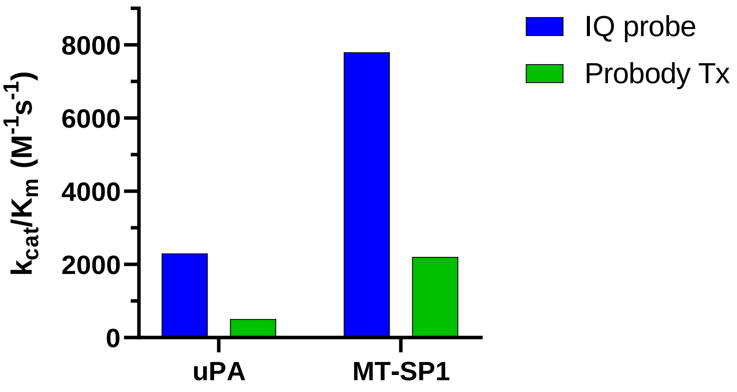

Figure S4. Substrate protease cleavability in an internally quenched (IQ) probe vs Pb-Tx format. k_{cat}/K_m of the S01 substrate was determined in the context of an internally quenched (IQ) probe versus a Pb-Tx for urokinase-type plasminogen activator (uPA) and membrane-type serine protease 1 (MT-SP1). Increased cleavability of the substrate in the format of a small peptide probe supports the impact of molecular scaffold and size on protease cleavability and thus potentially limited translatability to large molecules.

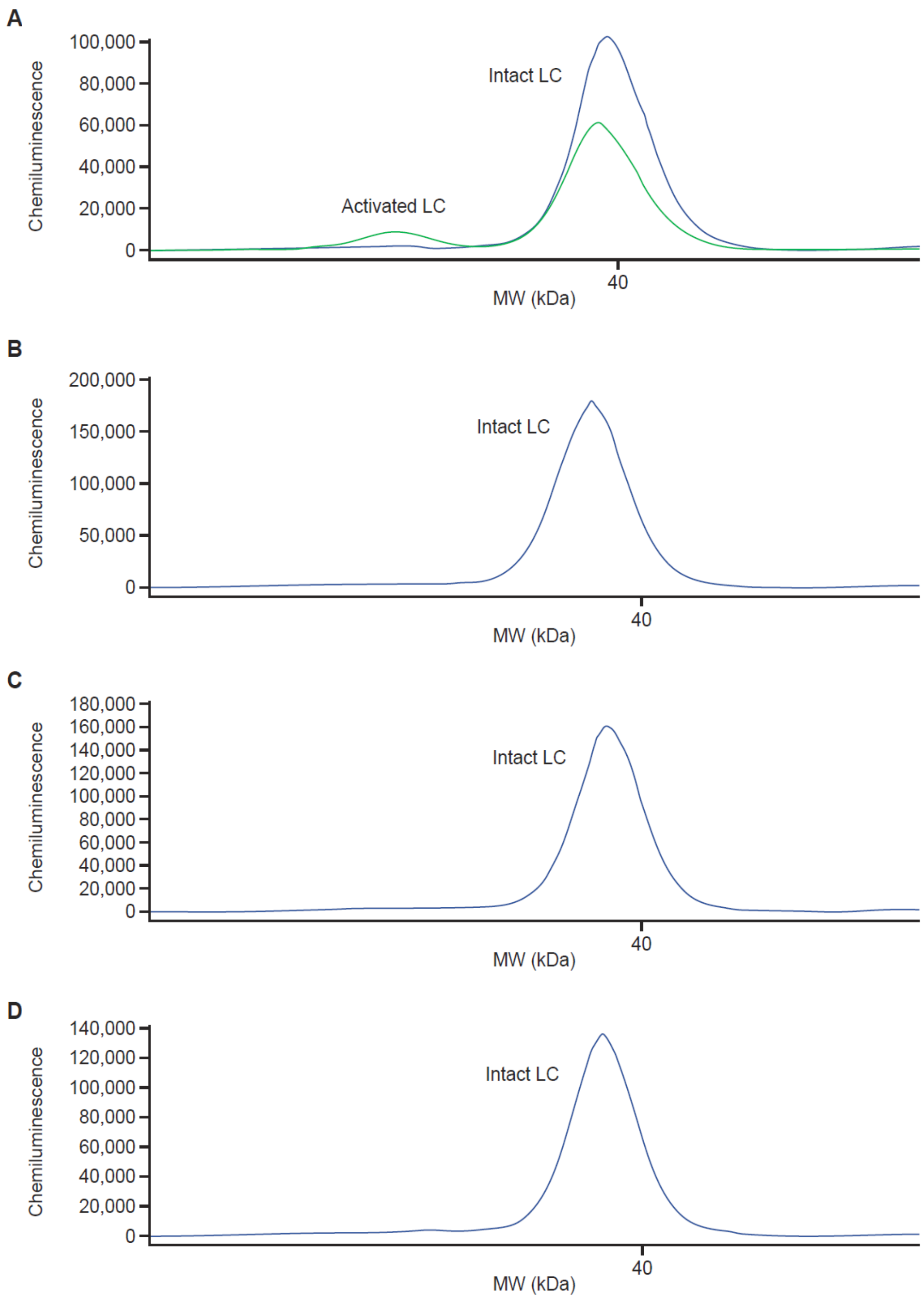

Figure S5. Electropherograms of CX-188 Pb-Tx incubated in plasma. (A) Membrane-type serine protease 1 (MT-SP1) activated CX-188 was mixed with intact CX-188 at a 20% ratio (green trace). Separation of an active and an intact peak (green trace) was apparent. This run was then overlaid with intact CX-188 (blue trace). (B) When incubated in normal plasma, CX-188 showed no activation, as indicated by the completely intact light chain (LC). CX-188 also showed no activation when incubated with (C) plasma from lung cancer patients or with (D) plasma from gastric cancer patients.
